# A Modified Wire Hanging Apparatus for Small Animal Muscle Function Testing

**DOI:** 10.1371/currents.md.1e2bec4e78697b7b0ff80ea25a1d38be

**Published:** 2016-05-02

**Authors:** Emma Hoffman, Steve J Winder

**Affiliations:** Department of Biomedical Science, University of Sheffield, Sheffield, United Kingdom; Department of Biomedical Science, University of Sheffield, Sheffield, United Kingdom

## Abstract

Wire hang tests are simple and cheap methods to assess muscle performance in small rodents, but do not always yield consistent results. We describe a simple wire hang apparatus that comprises a continuous rolling loop. Wire hang times measured using the rolling wire provide consistent and reliable data that more accurately reflect the output of a continuous physical effort. As such data obtained in mice using a rolling wire are more representative of the physical changes in the mouse muscle and less susceptible to individual mouse behaviour and differences in animal handling.

## Introduction

Rodents, and mice in particular are widely used as models of neuromuscular disease. Assessing muscle function in mice can however be difficult due to intrinsic inter-animal variation, mouse behaviour or the complexities of the procedure. Grip strength and rotarod measurements can be used, but require relatively sophisticated equipment and are not immune from the aforementioned problems 1. Direct physical measurement of the force of individual muscles can be recorded, but this requires a high level of technical competence and expensive equipment, furthermore these experiments are usually carried out under terminal anaesthesia, so cannot be used effectively in longitudinal studies 2. One simple low cost method is wire hanging, however this can suffer greatly from the effects of individual mouse behaviour. Wire hanging is best suited to measuring muscle coordination and endurance in mice and has been adopted as part of the comprehensive SHIRPA phenotypic testing regimen 3. Wire hanging is also particularly suited to dystrophic mouse models such as mdx 4. The precise type of apparatus used, how the test is implemented, coupled with individual mouse behaviour and the way the data are presented can have a significant impact on the result 5^,^^6^. Various refinements have been implemented in order to standardise the procedure and mitigate the effects of animal to animal variability 5. However differences in animal behaviour cannot easily be controlled for and with linear wire hang tests the number of times the animal reaches the end of the wire and has to be repositioned in the centre is an essentially random variable. We therefore sought to modify the traditional wire hanging apparatus to make a continuous wire thus eliminating the possibility of mice from reaching the end and therefore removing this aspect of variation in the data.

## Materials and Methods

Mdx and C57/bl6 mice were housed in the University of Sheffield animal facility according to national and international best practice guidelines for research using animals. All procedures were approved by the University of Sheffield Animal Welfare Committee and carried out under UK Home Office Project and Personal License (Animals in Scientific Procedures Act, 1986). In order to increase compliance and reduce animal stress, mice were handled using non-aversive methods as described previously 7^,^^8^. A standard linear wire hang apparatus was constructed, comprising a large plastic box 55cm long by 40cm wide by 35cm deep with a 2.5mm wire suspended in the top centre of the longest dimension,. A modified wire hanging apparatus was constructed using a 32cm circle of 2.5mm wire suspended on a pulley and placed so that the bottom of the loop of wire was positioned in the middle and at the top of an identical plastic box to above (Figure 1; detailed drawing and accompanying video of mice on the apparatus can be found in Supplementary Figure 1 and Supplementary Movie 1). We have termed this apparatus the rolling wire and hence the rolling wire hang test. A group of 11 mdx mice (32-41 weeks old) were then subjected on subsequent weeks to wire hang tests using either apparatus according to TREAT-NMD SOP 5 . For linear wire hang tests, the mice were placed in the centre of the wire with all four paws and a timer set running for 180 seconds. The timer is stopped when the mouse falls off the wire, or if it crawls along the wire and reaches the end. In either case, the mouse is then repositioned in the centre of the wire and the timer restarted, and repeated as many times as necessary up to 180 seconds. In all cases, mice fell and/or reached the end of the wire within 180 seconds and were repositioned at least 4 times. The individual times and number of reaches and falls were recorded. For the continuous wire hang apparatus, mice were positioned under the wire with all 4 paws in contact and three separate hang times recorded with a 30 second rest between each, with no limit on the duration of each individual period of hanging.

**The rolling wire hang apparatus.**
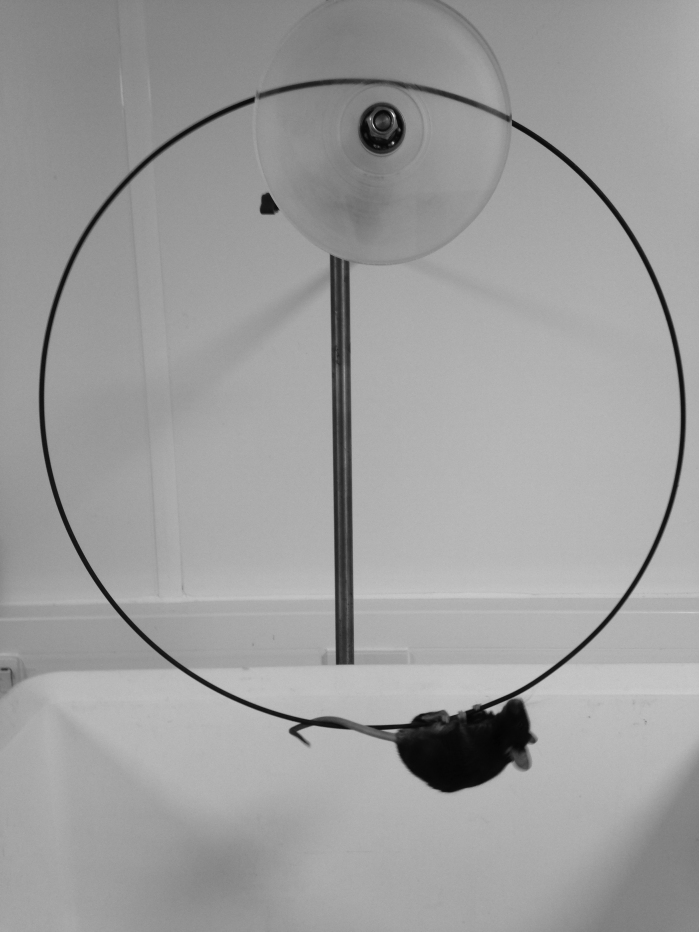
The apparatus comprises a flanged pulley to support and stabilise a 32cm wire hoop (see Supplementary Figure 1 for full details). The pulley is assembled on a steel bar that is clamped on a standard laboratory stand via a 2-way boss (partially obscured behind the pulley). The wire is positioned over a large box at least 30 cm from the base and 20cm from the sides to deter jumping. Bedding is placed in the bottom to break any fall. The mouse is positioned hanging with all four paws on the wire at the bottom. The time taken for the mouse to fall off is recorded. See also Supplementary Movie 1.

## Results and Discussion

The wire hanging test is based on the natural instinct of a mouse to avoid falling. The time that a mouse can hang for is determined by a number of factors, including the physical properties of the muscles, the number of limbs used and the weight of the mouse, as well as age and sex. Muscle disease such as muscular dystrophy, reduces hang times providing a rationale for using the test to determine the potential benefits of interventions. Mouse age and sex can be controlled in the experimental design, and weight can be accounted for in various ways in calculating the result. However, whether a mouse uses two or four limbs to hang on a wire is not easily controlled, nor is the direction and speed of movement, or whether the mouse falls off the wire when it reaches the end of a traditional linear wire hanging apparatus. Indeed distinctions between wire hanging using two limbs and grid hanging using all four limbs have yielded quite different results 6. Furthermore a mouse hanging on a grid can change its stance to use opposing muscles and/or vary the use of different muscle groups, making the gird hanging method a less consistent test of distinct limb muscles.

On a linear wire hanging apparatus, the number of reaches and falls also adds a variable duration rest factor into the experiment (Supplementary Figure 2). A mouse placed on a linear wire by its front paws may have the strength to lift its rear paws onto the wire and can also use its tail for extra balance, which greatly extends the hanging time. Nonetheless, on a linear wire using four paws, mice invariably go in one direction and are using the same stance and therefore the same muscle groups more or less continuously. Whilst most methods for wire hanging suggest starting the mouse with its front paws only on the wire, they do not exclude mice that then use their rear paws and tails to hold on 1^,^^5^. The only exclusion is a mouse that manages to balance on the wire, though this is relatively rare for mdx. Taking these factors into consideration we designed a perpetual linear wire that moved with the mouse so that the end of the wire was never reached, which here we shall term the rolling wire (Figure 1, Supplementary Figure 1 and Supplementary Movie 1). Thus if placed on the rolling wire with all four paws, a mouse will move relatively consistently in one direction using the same general stance and encounter no obstacles to its progression other than its own muscle performance and endurance. Consequently variation in numbers of limbs used, and the amount of rest time gained from falls and reaches is overcome (Supplementary Movie 1). Although we have routinely measured 3 consecutive hang times on the rolling wire, with 30 second rest between, the 1st hang time is almost always the longest (Figure 2) usually by a factor of at least 3, whereas with linear wires the longest hang time is more randomly distributed within the time interval measured (Figure 2). Because of the use of four limbs and generally a more consistent performance, mice hang times on the rolling wire are also longer than those on the linear wire (Figure 3). Therefore in order to make a fair comparison of mouse performance, and despite intrinsic difference in the length of the hang time between the two approaches we measured holding impulse in the same group of 11 mdx mice subjected to linear wire hanging and rolling wire hanging. Holding impulse (HI; hang time recorded in seconds multiplied by weight in grams) takes into account the generally shorter hang times of heavier mice, and can be represented as mean HI and maximum HI. Although the maximum and mean hang times are more than 5-fold longer for rolling wire, the degree of variability in the data is slightly lower for the rolling wire than for the linear wire (Figure 3).

**The distribution of maximum hang times in rolling and linear wire hang tests.**
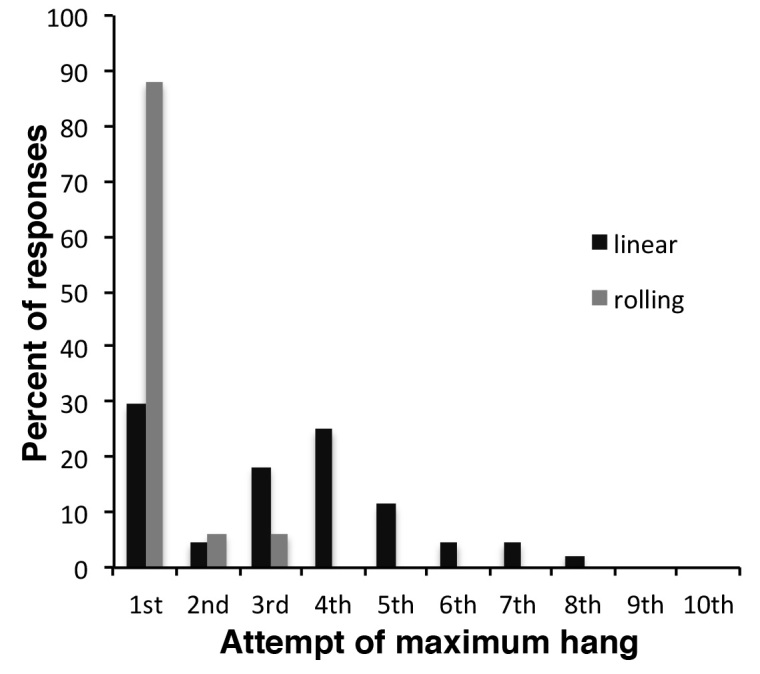
Mdx mice aged between 10 and 41 weeks were subjected to linear and/or rolling wire tests. The attempt during which the maximum time was achieved for any single mouse was recorded. In rolling wire hang tests (grey bar), the 1st hang was almost always the longest, whereas for linear wire hangs (black bars), there was a broader distribution of maximum hang times.

**Holding impulse scores for mdx mice in rolling or linear wire hang tests.**
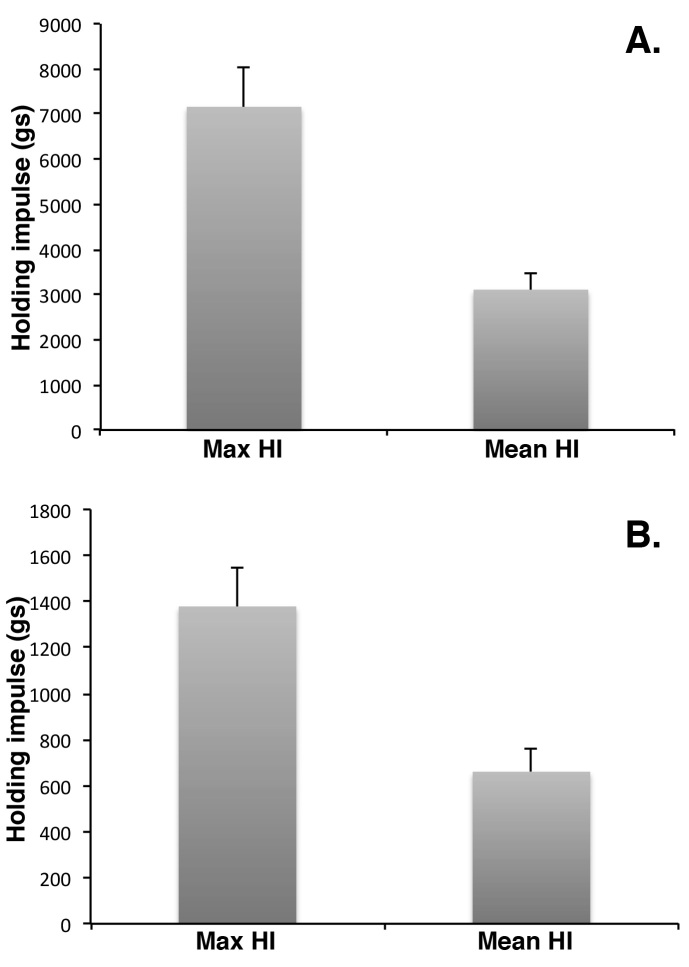
11 mdx mice aged between 32 and 41 weeks were subjected to rolling wire (A) or linear wire (B) hang tests. Data are recorded as the maximum holding impulse (HI) and mean HI. HI is hang time in seconds multiplied by animal weight in grams. Maximum rolling wire hang times are 5 fold larger than linear wire hang times due to the reduced frequency of falls. Data are mean ± SEM, n=11.

Depending on individual mouse behaviour, the number of reaches however can have a significant impact on the reliability of the data. Each time a mouse is repositioned, it is given a brief rest, so the more reaches in a given time will give the mouse proportionally more rest. Moreover, mice will often drop off the wire when they reach the end, adding to the rest period if they are not repositioned quickly. Consequently linear wire hang data do not and can not represent the full potential of the mouse for the task, as the mouse is unlikely to hang for as long as it possibly can because it will inevitably reach the end of the wire and either drop off or have to be repositioned. For healthier mice the situation becomes more acute, due to the mice being stronger and more agile, consequently they are likely to reach the end of a linear wire more frequently than animals with a greater disease burden. This does not arise with the rolling wire due to the continuous nature of the test and the ability of the mouse to hang and move uninterrupted. This is evident from the frequency distribution of longest hangs shown in Figure . On a rolling wire >90% of first hang times are the longest, whereas for a linear wire the longest hang is more dependent on behaviour and the effect of tiring after repeated reaches and repositioning (Figure 2, Supplementary Figure 1). Furthermore, the rolling wire hang test can easily detect differences between mdx mice and wild type mice, as shown in Supplementary Figure 3. Thus the rolling wire test represents better the continuous physical output of the animal making it a closer correlate to the 6 minute walk test long used as a test of cardio-respiratory function 9 and more recently adopted as an end-point for assessing muscular dystrophy^10^.

We have designed and validated a modified wire hang apparatus that yields a consistent and physiologically more meaningful output than the more traditional linear wire hang apparatus. It is simple to construct, easy to use and provides reliable results when tested with mdx mice. The output from the rolling wire hang test is a more rigorous test of mouse muscle performance than the linear wire hang test.

## Supplementary Figures

**Figure:**
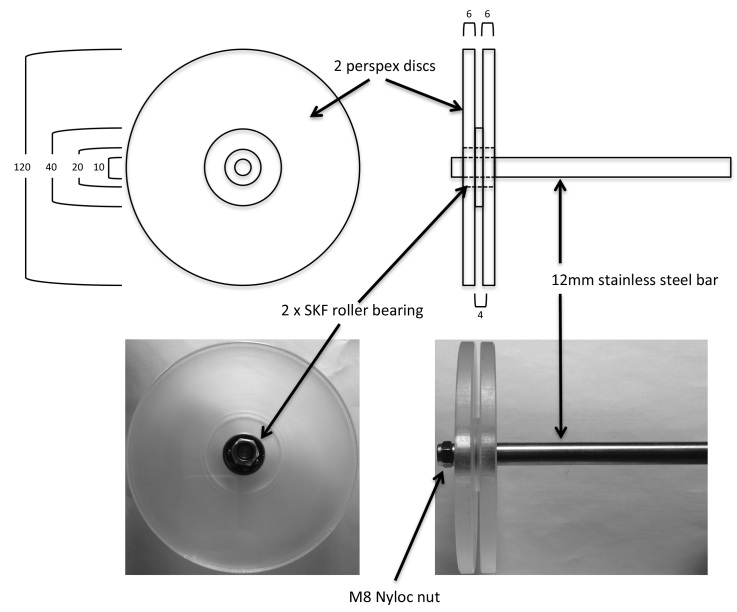
**Supplementary Figure 1.** Diagram of rolling wire apparatus components and construction.

**Figure:**
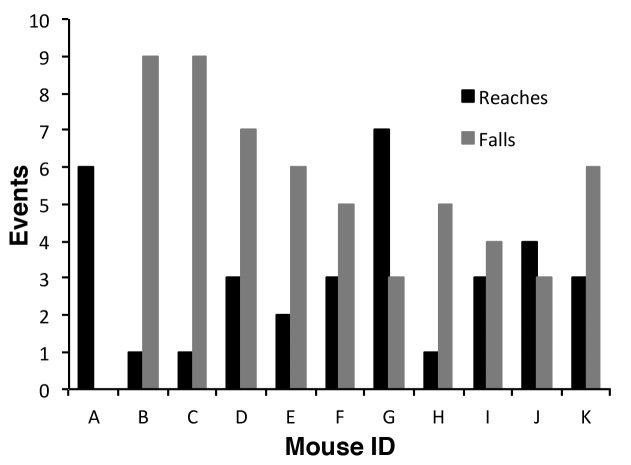
**Supplementary Figure 2.** On a linear wire hanging apparatus, the number of reaches and falls adds a variable duration rest factor into the experiment.

**Figure:**
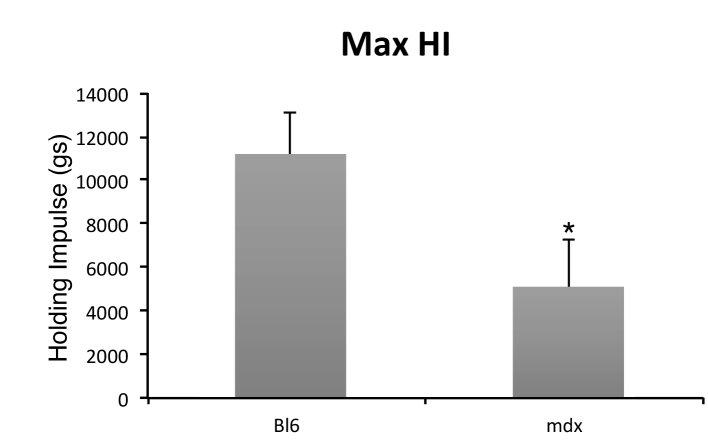
**Supplementary Figure 3.** Comparison of wild type and mdx using the rolling wire hang test. A group of eight 12 week old mdx mice were compared with five 12 week old normal C57bl6 mice using the rolling wire hang test. Hang times using the rolling wire test, calculated as maximum holding impulse, were significantly different between the two groups, * p=0.013.
